# Taping things together: exploring a group supervision method in a healthcare organization

**DOI:** 10.3389/frhs.2026.1744726

**Published:** 2026-02-13

**Authors:** Marie Thegerström, Ingrid Schéle, Erik Lundkvist

**Affiliations:** 1 Occupational Health Services, Region Kalmar County, Kalmar, Sweden; 2 Department of Psychology, Umeå University, Umeå, Sweden

**Keywords:** group supervision, healthcare professionals, occupational health services, taping method, workplace health intervention

## Abstract

**Introduction:**

Healthcare professionals face high levels of stress and emotional demands, highlighting the need for supportive interventions. This study aimed to explore the experiences of healthcare staff participating in group supervision using the creative method of “taping,” which involves arranging figures and symbols to visualize and reflect on complex work situations.

**Methods:**

This qualitative study included fourteen participants divided into four healthcare work groups. Each group engaged in five to six supervision sessions, after which participants took part in individual interviews. Data were analyzed using thematic analysis. The study was approved by a local ethical review board.

**Results:**

Three main themes were identified: (1) the significance of group supervision for the workgroup and work environment, (2) enhanced recognition and understanding of emotions and behaviors, and (3) support for behavioral change. Participants described increased trust, improved communication, and greater emotional insight. The taping method was perceived as particularly effective in clarifying challenges and fostering shared understanding through visual representation. It also encouraged collective and individual reflection, goal-setting and strategic action, associated with self-regulated learning.

**Discussion:**

The findings suggest that group supervision using the taping method can enhance well-being, strengthen team cohesion, and promote professional development among healthcare staff. This creative approach appears to facilitate emotional awareness and collaborative learning. Further research with larger and more diverse samples is needed to examine the method's broader applicability and long-term effects.

## 1 Introduction

The work environment for healthcare professionals in Sweden has long been trying, with high workloads and stress posing risks to employee health and retention, as many leave the profession (1). During the COVID-19 pandemic, healthcare systems faced unprecedented pressure, further exacerbating these issues (2, 3). To address these challenges, workplace interventions for healthcare professionals must promote sustainable work conditions (4). Occupational health services play a crucial role in offering initiatives that improve the work environment. Research that evaluates such interventions is particularly important (5). This study will explore the feasibility of an intervention method to address the challenges that healthcare professionals face in their work environment, and describe their experiences thereof, using interviews to gain in-depth understanding.

Group supervision is a work process-promoting intervention aimed at improving job satisfaction, professional competence, and tolerance of workplace stressors, as well as facilitating emotional management in challenging situations (6–8). Such interventions also support reflection and recovery, encourage collaboration and shared responsibility in the workplace (9), and help prevent compassion fatigue and burnout (8).

The reflective process in group supervision may help participants recognize their emotional responses and reactions in challenging situations. Research on group supervision in human service organizations has identified several key dimensions that contribute to beneficial worker outcomes, including task assistance, social and emotional support, and the quality of interpersonal interaction between supervisor and supervisee (10). These dimensions are primarily enacted through verbal and relational processes. This, in turn, can foster psychosocial understanding, enhance relational skills at work, and aid formation of strategies for managing complex daily tasks (10, 11). However, differing perspectives or goals among participants can cause fragmentation in the process, and varying levels of experience and knowledge may complicate efforts to meet everyone's needs (10). Integrating creative methods into group supervision is a promising approach to address these challenges. Creativity enhances problem-solving and emotional expression, fostering innovation and well-being within teams (12). Creative approaches also deepen the reflective process, helping participants engage more fully with their emotions, behaviors, and professional challenges (13).

One creative approach is taping, originally developed within child-centered family therapy. It uses figurines and symbols to represent relationships, emotions, and challenges, to provide a framework for reflection and discussion. Taping is adapted for use in group supervision and serves as a tool to enhance reflection and facilitate understanding of complex professional situations (14, 15).

Figurines and symbols are selected and arranged by the supervisees, who assigns meaning to each element based on their personal interpretation. Taping offers a “helicopter view,” enabling the supervisee to concretize a situation for themselves and others in the group. This can facilitate personal growth and learning while also supporting development within the professional role. Taping can stimulate learning by offering a tangible, visual representation through figurines and symbols, which reinforces both memory and understanding (14, 15).

Taping could also support learning by encouraging supervisees to actively engage with their challenges and experiences. This aligns closely with the principles of self-regulated learning (SRL), a framework that emphasizes personal responsibility, goal-setting, and strategic action during the learning process. Whereas taping serves as a tangible, creative medium for reflection, SRL offers a theoretical lens to understand how individuals can take ownership of their professional development within a supportive, interactive context. Together, these approaches create a powerful synergy for fostering insight, growth, and effective problem-solving in workplace settings.

SRL is relevant in workplaces where challenges like procrastination and unclear planning can hinder performance and growth (16, 17).

In group supervision, participants are encouraged to take responsibility for their own learning and development. Taping, as a creative and reflective method, aligns with SRL principles by helping participants visualize challenges, set goals, and evaluate strategies. The collaborative and social nature of taping mirrors the contextual and interactive elements emphasized in SRL, fostering personal insight and professional growth in healthcare settings.

Given the demanding conditions within the healthcare sector, it is essential to create and maintain a positive work environment for healthcare professionals. Occupational health services, a key resource in promoting workplace health, need to provide evidence-based interventions of high quality. Group supervision is one such intervention offered by Swedish occupational health services to support healthcare professionals in managing work-related challenges, enhancing reflection, and fostering collaboration.

The aim of this study was to explore healthcare professionals’ experiences of participating in group supervision using taping. Additionally, the study sought to examine if taping was perceived as helping in daily work and relationships with colleagues and managers.

## 2 Methods

### 2.1 Study population

Interviews were conducted with healthcare professionals who had participated in taping group supervision. The four healthcare supervision groups next in line for occupational health services’ supervision at the start of this study were invited to participate, three of which did. Each consisted of 4–6 participants, for a total of 14 participants. The participants worked as nurses, assistant nurses, and coordinators in the healthcare sector. All but one of the participants were female, and their professional experience ranged from newly employed to over 20 years in the field (more information in Table 1). The group that declined participation did so due to a high workload. We have not identified any systematic differences between the participating and non-participating groups, and as a result, the findings are mainly relevant to these participants and the organization to which they belong.

**Table 1:** Participant characteristics.

Age		Gender		Education	
35 years and younger	3	Female	11	High school	4
36–50 years	5	Male	1	Bachelor	5
50 years and older	4			Master	3

The study followed the ethical guidelines of the Swedish Research Council (18) and received advisory ethical review Southeast Swedish Ethical Committee (ref. no. 850-2022) where they saw no issues where an ethical application to the Swedish Ethical Review Authority was needed. Participants and their managers received detailed information, provided written consent, and were assured that participation was voluntary and without consequence for their supervision experience.

### 2.2 Research process

Each of the three groups completed 5–6 supervision sessions, held every 2–3 weeks for 2 h per session. After the supervision period ended, the 12 participants who had agreed to this were interviewed. Semi-structured interviews were conducted by an external facilitator and a psychologist from occupational health services, as the first author had supervised the groups. The interviews lasted 20–40 min.

### 2.3 Analysis

The data were analyzed using thematic analysis (19, 20). Once the transcription of an interview was completed, the entire interview was read through to gain an overall understanding. The first author then identified meaningful segments within the text and assigned around 270 initial codes.

The coding was conducted inductively, meaning that codes were derived from the data rather than from a predefined framework. Codes that appeared to share meaning or address similar aspects of the participants’ experiences were then grouped together into potential subthemes. This was made from printed interview where codes where color coded and cut out individually, sorted to themes and thereafter organized in a word table.

The process was iterative, with ongoing comparisons between the data and the emerging themes. When subthemes and themes began to take shape, these were discussed with the co-authors, who reviewed the coherence between codes, subthemes, and themes and offered alternative interpretations. This collaborative process led to refinements in theme labels and structure.

The figures presented in this study were developed inductively during the analytic process, emerging as the relationships between codes and themes became clearer. Rather than being constructed retrospectively for illustration, they were used as analytical tools to explore and visualize how participants’ experiences connected to the supervision process and to each other.

Managing the risk that the first author's prior understanding, stemming from their role as a facilitator in the supervised groups, might influence the analysis, a descriptive approach was adopted, sticking close to the participants’ own accounts (20). Furthermore, the first author continuously and rigorously reflected on their assumptions and preconceptions throughout the project, as emphasized by Ho et al. [(21), p. 1760].

### 2.4 Trustworthiness and reflexivity

We followed Braun's and Clarke's (20) guidelines for thematic analysis, which emphasize transparency and reflexivity in the analytic process. Themes were developed systematically, supported by participant quotes to illustrate their relevance. Recognizing the active role of the researcher in meaning-making, we adopted a reflexive stance, acknowledging how our backgrounds shaped both data interpretation and theme development. The first author, experienced in the taping method and familiar with the supervision groups, balanced insider knowledge with critical reflection to maintain analytic rigor. This was done for instance by using independent interviewers. To further enhance trustworthiness, the third author acted as a critical friend, challenging interpretations and encouraging deeper analytical engagement. Rather than seeking objectivity, we embraced a position of transparency and made researcher influence explicit, to strengthen the study's credibility.

The coding was conducted in several stages. Initial codes were continuously reconsidered as the first author reflected on questions such as: “Am I distinguishing here between the participants’ statements and my own experiences of the group?” Coding decisions and interpretations were discussed with co-authors, serving as an external check and helping to explore alternative interpretations. This strengthened the trustworthiness of the analysis.

Generative AI (ChatGPT-4o and Academic Assistant Pro) was used to support translation from Swedish to English and for language editing in this study. The AI tools assisted in refining clarity and coherence while ensuring alignment with academic conventions. All outputs were critically reviewed and revised by the authors to maintain accuracy and integrity.

## 3 Results

**Figure 1:**
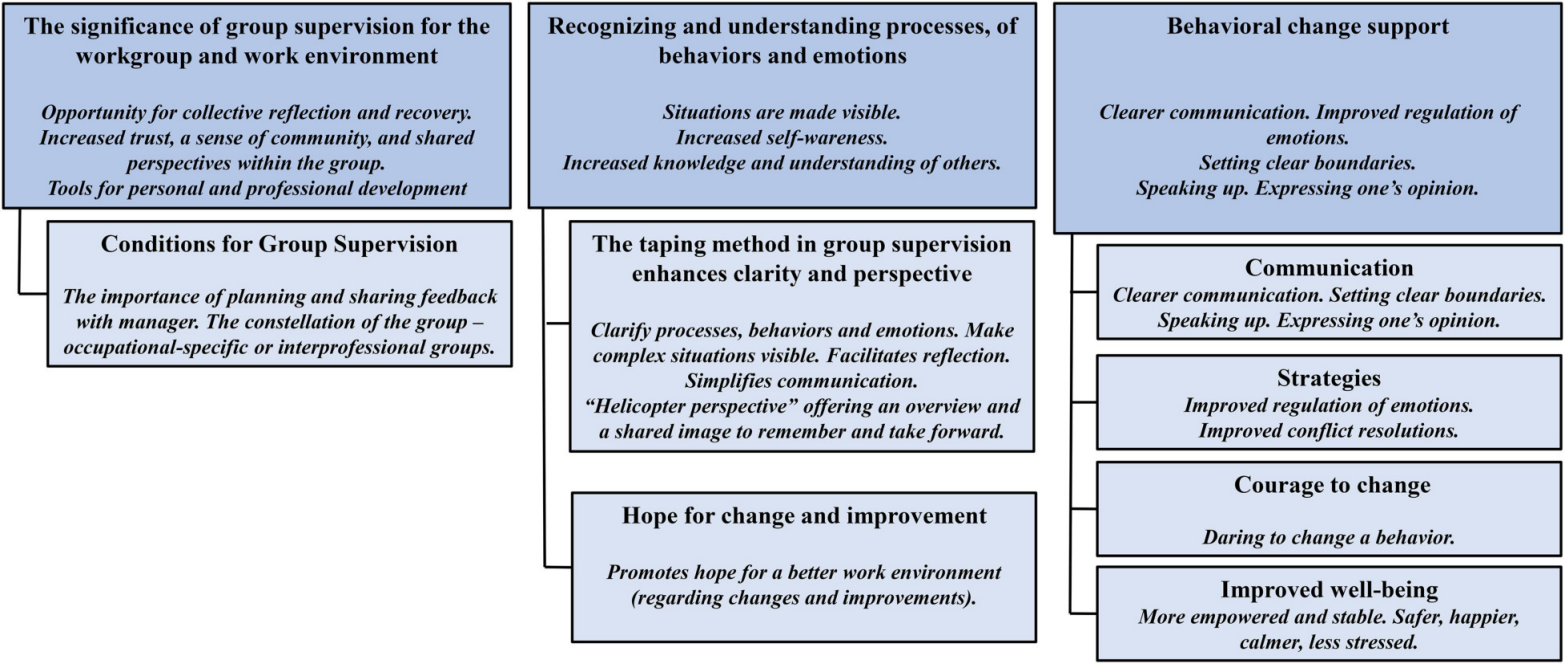
Overview of themes and subthemes.

Three main themes have been identified: (1) The significance of group supervision for the workgroup and work environment, (2) Recognizing and understanding processes, behaviors, and emotions, and (3) Behavioral change support (see Figure 1). Situations involving colleagues discussed during group supervision were described as experiences and lessons that could also be applied to interactions with patients, and vice versa.

### 3.1 The significance of group supervision for the workgroup and work environment

The participants identified several positive aspects of participating in group supervision. The opportunity for collective reflection was highlighted, with many participants wishing for such sessions to be integrated into their everyday work.

“… You hear what others find challenging, and you get a chance to talk about what you feel, think, and experience, which you might not always be able to do so openly at work.”

Collectively reflecting on challenging situations was described as beneficial for articulating and understanding individual emotions and experiences, as well as fostering a sense of shared perspective and unity within the workgroup.

“We’ve gained more trust in each other. … We support one another…and we remind each other of the things we’ve learned during this group supervision. We have the same mindset, we share the same perspective, and we want the same thing, and we talk about that together almost daily. … It can be small things, and it can be big things.”

The participants described how group supervision increased mutual understanding, which they found empowering and contributed to increased support and a stronger sense of safety within the workgroup.

#### 3.1.1 Conditions for group supervision

Some participants described advantages of group supervision for one professional category only, noting that it fostered a specific sense of safety and strengthened their professional identity. The opportunity to share professional experiences may have created an implicit understanding that enhanced psychological safety. Shared frames of reference may also have facilitated recognition, making it easier to appreciate others’ perspectives. Others, however, saw benefits in interprofessional supervision groups, involving all the occupational categories at the workplace. The purpose of mixed groups could be to enhance cohesion and foster a shared perspective within the workplace.

“There are definitely some fences between the professional groups, and you can tell that we’re different. So, sure, it probably wouldn’t have been as open at the beginning, but I think that’s the kind of thing that could build more of a team spirit. Supervision together might have helped remove those fences between the professional groups. In that way. I think we would eventually have realized that we probably shared the same problems, that all the professional groups were experiencing the same issues.”

The participants also emphasized that group supervision should include opportunities for meetings with managers. Such meetings would provide a platform to relay insights from the group, which could potentially benefit the organization's ongoing development and improvement efforts.

### 3.2 Recognizing and understanding processes, behaviors, and emotions

The participants described how, during supervision sessions, they selected situations from their work that were perceived as difficult or challenging and that they wished to explore and understand more deeply. These situations could involve, for instance, dealing with unpleasant interactions or managing strong emotions, such as frustration, fear, or anger, which might hinder a professional approach toward patients or colleagues. Participants noted that explicitly talking about emotions or seemingly everyday events often led to new insights about processes and behaviors they had not been aware of before.

“… It became clear to me, what I had felt but couldn’t put into words. It was frustration. I had nothing to do with it but kept getting tossed back and forth. Why should I take on that role? That became very clear here. I’ve definitely gained some insights I didn’t know I needed. Nothing has changed at work, but I have that insight. And we’re talking about it a bit more.”

Participants also noted that the situations discussed, reflected upon, or organized during the supervision sessions contributed to greater self-awareness.

“The positive part has been finding tools to understand other people’s behaviors and reactions…how people are, how they can influence others, and what they want with their behavior. Understanding that…learning to handle it and distance oneself from it. Learning to understand your own feelings when you can’t quite pinpoint what they are…those ‘aha moments,’ in different areas, have been so good. That’s probably been the best part. …there have been emotions; I’ve cried there. That’s how good it’s been, because so much has come to the surface.”

Participants highlighted that the supervision process helped them gain clarity and a better understanding of their own emotions and behaviors, both in general situations and in particularly challenging ones. This also contributed to a deeper understanding of how others might feel, act, and what might underlie their behavior. The increased knowledge of themselves and others, along with how interactions with others are influenced by these factors, was described as providing tools for handling future challenging situations.

#### 3.2.1 Enhanced clarity and perspective

The participants described how the taping method helped clarify processes, behaviors, and emotions during group supervision. They emphasized that the method also facilitated an understanding of the complexity of situations: “*…it becomes so crystal clear what you see in front of you.”*

Although some participants noted that finding suitable symbols to represent emotions and experiences—such as animals—could sometimes be challenging, they highlighted several advantages in constructing a scene compared with merely discussing a situation verbally. For instance, when a situation was set up on the table using figurines, animals, and other symbols, the scene became visible to everyone, providing a shared visual reference for reflection. This could also make it easier to understand others by “seeing” their thoughts and feelings.

“…even if it’s not a real conflict, but there’s some tension and difficulty, it’s really important to bring in the emotions, because they’re what makes it hard. Using the animals to sort through it, what emotions are present? Why are we afraid of that emotion or why do we want to withdraw? …we saw a clear map of what was happening in the room. In that way, I think it was a really good method, it was clear. …we all had the same picture. Otherwise, when you’re just talking, you probably see four different versions of the scenario.”

The taping method also provided a “helicopter view,” offering an overview where multiple perspectives could be visualized simultaneously. Being able to see multiple perspectives at once may have facilitated understanding of both one’s own and others’ actions. This overview was said to bring clarity and create a shared image to carry forward, serving as a memory aid or a new visual language that simplified communication within the workgroup after the supervision sessions.

“We refer back to those situations and the scenarios we built, like, “Today I had that dinosaur on my shoulder again.” Then everyone understands…that you feel irritated. … The other day, someone said, “Today I got that damn rock in my lap.” I know exactly what she means when she says she’s got a rock in her lap. Yes, of course. It’s like that. The symbols became very clear, and everyone understands what it means when you’re sitting there with a big rock in your lap. It becomes extremely clear.”

#### 3.2.2 Hope for change and improvement

The participants described how group supervision made emotions, behaviors, and processes visible—whether related to themselves, others, or dynamics within the workgroup or workplace as a whole. This was perceived as both encouraging and relieving. It was described as a relief to collectively identify the sources of frustration or address issues that felt vague or difficult to grasp. According to the participants, this process fostered hope for a better work environment.

“Since I’m conflict-averse, for me, it’s about not avoiding everything all the time and daring to stay in the situation when it gets a bit uncomfortable. That’s what I see as a process, because it feels hard just thinking about it. But I really want to be able to…and it’s not about reacting the way one might, by getting angry, but rather about gaining an overview and naming the situation. And being able to express it calmly. …you get to reflect a lot about yourself, about who you are as a person, during this supervision.”

“I think we were all kind of, well, pretty satisfied with the things that came up and that we were able to discover. That maybe there are solutions that aren’t completely unrealistic or incredibly expensive, but rather simple things we could start with… just thinking in other ways.”

### 3.3 Behavioral change support

The participants described how group supervision contributed to the ability to modify their own behavior, for instance, to foster better and more sustainable relationships or to feel secure and empowered thanks to having effective tools and strategies for managing difficult or challenging interactions with others. However, some participants stated that group supervision had little to no impact on their behavior. This may be due to differences in previous experiences of reflection or need for a change in the work situation. One's own experiences of psychological safety within the group may also have played a role. This suggests that experiences of group supervision may vary between individuals, depending on the context and personal factors.

#### 3.3.1 Communication

The participants described how group supervision enhanced their reflections and awareness regarding how they communicated and expressed themselves—an insight they felt led to subsequent changes in their behavior within the workgroup.

“I communicate better. Before, I would just keep getting annoyed about things, but I wouldn’t say anything. Then it would build up to a certain point, and I’d feel terrible, I couldn’t sleep. Now, by setting clear boundaries and being more direct in my communication, I refer problems to the right people. …Previously, it was like, “Oh, they’ve done it wrong again,” and I’d just get annoyed and grumpy, but the person didn’t even know why I was upset. So, it’s better to be upfront. I think clarity is what I’ve learned.”

The participants also explained that as they gained greater trust in their emotions and expertise; they felt more confident than earlier. They now dared to speak up and express their opinions and were more conscious of how their communication and actions influenced interactions and relationships. As a result, they described their communication as clearer and more objective. The sense of safety within the group may have created a space for practicing new ways of expressing oneself. The opportunity to have one's behavioral patterns and emotions visualized may have contributed to increased self-awareness.

#### 3.3.2 Strategies

The participants described how they developed new strategies during group supervision that they have since implemented into their work. For instance, they noted an increased ability to remain calm and that they were less prone to frustration compared with before participating in the supervision sessions.

“Above all, being able to calmly and kindly say “I feel hurt when you say this.” … And speaking at a calm pace and in a calm voice. Not letting yourself get worked up or angry if the other person is upset, because it doesn’t help. … Not getting worked up and staying calm, that's probably the clearest change. … This thing about thinking first and then speaking, to think through what you’re going to say before you say it. Instead of the other way around. That’s good.”

Improved emotional regulation was highlighted as a key strategy, particularly in situations where a conflict was beginning to escalate. Participants described how consciously maintaining calm allowed them to think through the situation, reflect on their emotions, and decide how to act and communicate effectively. Understanding the emotions underlying both their own behaviors and those of others was considered crucial for maintaining composure.

#### 3.3.3 Courage to change

According to the participants, courage was required to change behaviors, especially in situations associated with fear or discomfort, such as daring to stand firm and express one's opinions. Tools and support gained from group supervision were described as helpful in facilitating behavioral change.

“…[the group supervision] has helped me and strengthened me to dare to speak up. Where I previously wouldn’t have spoken up and would have stayed silent, I now dare to stand up for myself: “No, I think this has gone too far.” … I feel like I can voice my opinion, say what I think, and point out when something is wrong.”

“To dare to stand by what I say, and to express my opinions or how I want to work. … Not just be a little gray mouse that sits there and takes everything from everyone else, but…

Interviewer: The courage to stand up for yourself?

Participant: Mmm… I think that's probably the biggest thing.”

Participants explained that group supervision brought an awareness of the need to change their strategies, for instance in handling unpleasant behavior. However, they noted that this kind of change, requiring courage, often took time and differed between individuals.

“No, I haven’t [made the change] yet, but I know I need to take a firmer stand. It’s not like I’m treated terribly all the time, but sometimes I still tend to avoid confrontation even though I know I probably shouldn’t…because, again, we’ve talked a lot about how you can address it in different ways. Ask me again in a year.”

In some cases, participants described how problems identified within the workgroup were found to have causes beyond individual behavior and might instead stem from systemic or organizational factors.

“…you can see that the cause of the problems in the staff group doesn’t actually come from the staff but is largely at the management level. … It’s always good to realize that. It’s harder to do something about it, though.”

#### 3.3.4 Improved well-being

The participants highlighted that group supervision improved their overall well-being, making them feel more confident and empowered, both in their professional roles and as individuals. They described how their increased ability to remain calm and articulate their opinions contributed to better relationships with others and a reduced sense of stress.

“I solve problems here at work instead of taking them home, sleeping poorly, and not being rested for the next day, which just creates a vicious cycle. I’ve learned to take a step back, and maybe I don’t need to say everything in every situation—those involved can talk to each other instead. I’m much more stable as a person. … I set boundaries much better, both here and in my personal life, and in that way I work more efficiently, get more done, and am happier. Honestly, yes.”

“I feel much calmer; I don’t get as frustrated. And if I do get frustrated, I might say it in a different way, so I don’t get as angry. … And it doesn’t take up as much energy as when you feel the need to get angry, and I don’t take it home with me as much. … Overall, I think it’s easier within the workgroup.”

According to the participants, being able to speak up at work reduced the tendency to dwell on work-related events at home. This led to improved sleep, which they described as having a positive impact on their mood.

## 4 Discussion

This study examined how taping group supervision is perceived by healthcare professionals. The results highlight that this intervention can provide opportunities for reflection and recovery. It was perceived to contribute to increased trust, a sense of community, and shared perspectives within a (work)group. Participants further described how group supervision fostered a greater understanding of one another while offering tools for personal and professional development. Group supervision might also improve overall well-being, likely reflecting a general improvement in the social work environment. However, these changes cannot be directly attributed to the taping method and should be understood as participants’ subjective experiences within the scope of this study. These findings align with previous research on group supervision (9, 11). As such, group supervision shows promise as an intervention that could help improve the work environment for individuals and teams in healthcare settings.

The findings also align with previous research on supervision, which emphasizes task assistance, social and emotional support, and interpersonal interaction as key supervisory dimensions linked to beneficial worker outcomes (10). The present study suggests that these processes can be strengthened when complemented by creative and visual approaches. Through taping, participants’ experiences and challenges became visible in ways that verbal dialogue alone may not achieve.

The use of figurines and symbols helped participants make professional challenges tangible, structure them, and explore them in greater depth. This extends the work of Hafford-Letchfield and Huss (13), who argued that creative approaches deepen the reflective process in supervision. The present findings suggest that supervision and workplace learning can benefit from incorporating creative methods that manifest visually, particularly in complex and emotionally demanding situations.

**Figure 2:**
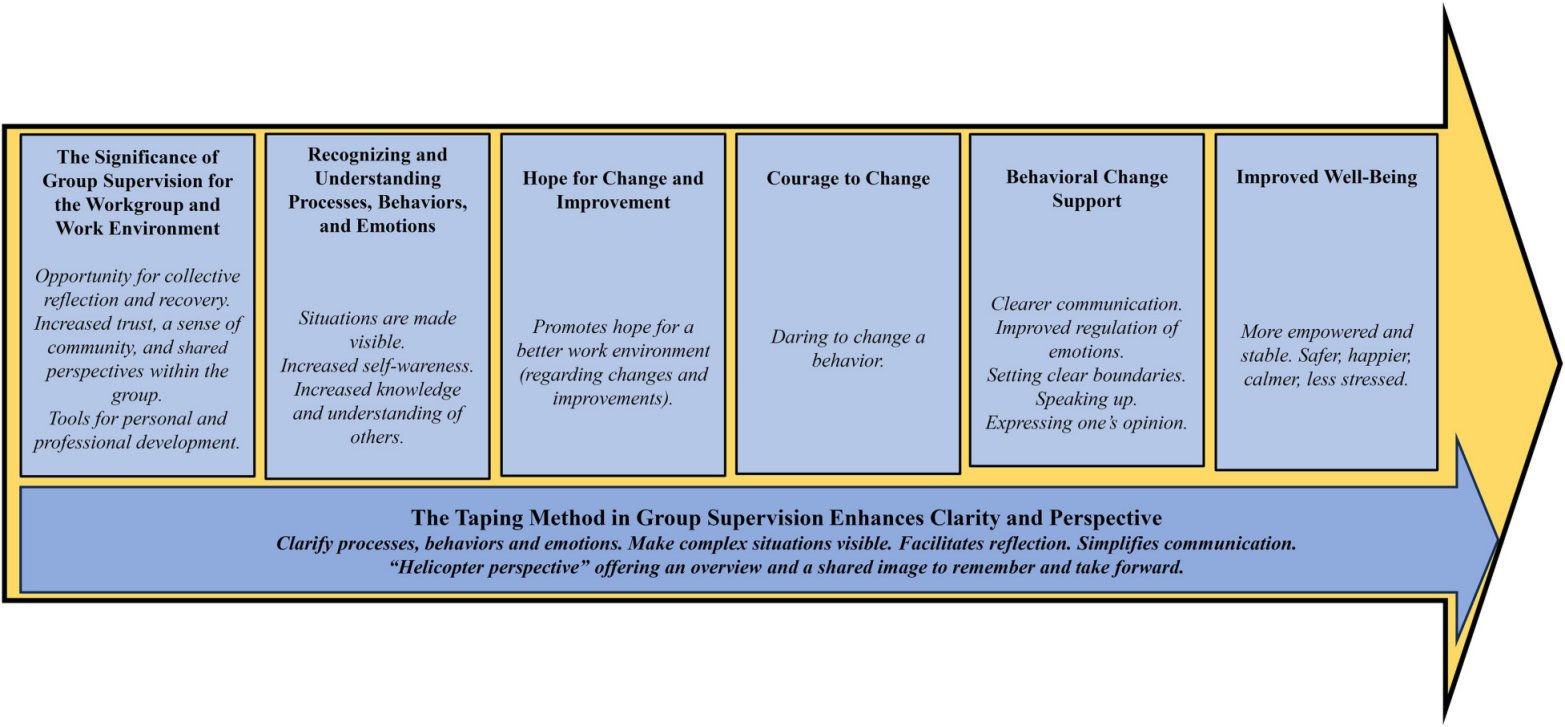
Group supervision using the taping method—a description of the supervision process based on themes emerging from the results.

The taping method used in this study's group supervision sessions was described by participants as a valuable tool, particularly for clarifying dilemmas, challenges, and processes, as well as emotions and behaviors. This finding is consistent with previously published case studies on taping in supervision (14, 15). Healthcare work often involves managing difficult, complex, and emotionally challenging situations that require clarification, understanding, and learning [see (14)]. Taping was described by participants as an effective way to illuminate emotions and behaviors, suggesting that it could be particularly suitable for healthcare professionals in group supervision. The taping method also appears to enhance participants’ processes of learning and change. Figure 2 illustrates how the taping method, based on participants’ descriptions, acts as a guide throughout the supervision process, from the sessions as such to changes in behavior and well-being. When taping is used, it initiates a creative and emotional process that activates multiple senses. According to Pakzad (22), this can enhance learning.

In addition to helping participants understand emotions, the use of figurines, animals, and symbols in taping also facilitates the creation of a shared image when a situation is set up, which the participants highlighted as an advantage compared with verbal descriptions alone. A shared visual representation can enhance not only learning but also memory (14). The memory of the image created during supervision may persist over time, deepening the learning process, particularly when there is an emotional connection (22). This process could stimulate both implementation and maintenance of behavioral changes, indicating that the taping method may support the development of self-regulated learning.

When taping contributes to a mutual understanding of a work situation, it may also positively affect the work environment. A shared perspective can strengthen the sense of community within the workgroup and facilitate the creation of collective strategies for change (23). Participants described how the visualization of situations using animals and symbols could evolve into a shared language, clarifying and simplifying communication within the team. Taping may also support participants in articulating emotions and addressing challenging situations outside of the group supervision setting (14). This dual function of enhancing supervision and fostering self-regulated learning aligns with findings from previous research (16, 17).

Group supervision with the taping method thus appears to combine elements that enhance learning. Participants described how figurines and symbols evoked previously unacknowledged thoughts and emotions. This emotional component of supervision, combined with the ability of the constructed scene to strengthen memory, supported the learning process [see (22)]. Learning is particularly crucial for healthcare professionals due to the demands of rapid adaptability and efficient performance under a high workload. To meet these demands, healthcare staff must develop self-regulated strategies that are continually reassessed and adapted to a changing environment [see (16)].

The findings from this study suggest that group supervision with the taping method can help participants recognize and understand their own emotions and behaviors and those of others, providing opportunities to test and evaluate alternative strategies. These strategies can support professional development and help maintain and improve relationships with colleagues and patients.

The findings related to behavioral change were disparate, with some participants reporting little to no impact. A closer examination of these accounts suggests two patterns. First, some participants described behavioral change as an ongoing process that required time and courage, indicating that change had begun but was not yet fully realized at the time of the interview. As one participant noted: “Ask me again in a year”. Second, some participants came to understand that the challenges they faced were rooted at the organizational or management level rather than in their own behavior. For these participants, the supervision process provided valuable insight, but the problems identified were not amenable to individual behavioral solutions. This suggests that the absence of reported behavioral change does not necessarily indicate a lack of impact; rather, the supervision may have contributed to a clearer understanding of the situation, including recognition of the limits of individual agency.

The results suggest that taping in group supervision may support aspects of self-regulated learning, particularly the self-reflection phase (16, 17). Participants described how the taping process helped them recognize and articulate emotions and reactions that had previously been difficult to verbalize. As one participant expressed: “It became clear to me—what I had felt but couldn't put into words.” This type of insight reflects the self-evaluative processes central to self-regulated learning. The visual and hands-on nature of taping also appeared to support self-observation during the sessions. By constructing and viewing a concrete representation of a situation, a “helicopter view”, participants could monitor and adjust their understanding in ways that may be more difficult through verbal reflection alone. Furthermore, the shared images created during supervision served as reference points that participants returned to in their daily work, suggesting that the method may support the transfer of learning beyond the supervision sessions. A notable aspect of these findings is that such outcomes appear achievable after just 5–6 group supervision sessions, indicating that taping may offer an accessible approach to supporting self-regulated learning in workplace settings.

### 4.1 Strengths and limitations

This study has several limitations that may have influenced the results. The first author's role as a facilitator for the supervision groups created preconceptions that may have affected the study's objectivity and credibility. To mitigate this risk, a descriptive analysis was chosen, and the thematic analysis was conducted twice to verify the stability of the themes and subthemes.

Another limitation is that all participants were drawn from the same organization, within a healthcare context, and the majority were women (13 out of 14). These factors may have influenced group dynamics and the nature of emotional expression during the sessions. Future research should examine whether similar findings emerge in other organizational contexts and in groups with different gender compositions. However, a strength of the study is the diversity among participants, who represented a variety of workplaces and professions and occupations within healthcare. The participants also varied in age, professional experience, and educational background. It is a strength that the study involved groups already scheduled for group supervision through occupational health services, as this meant that the study examined the occupational health services’ regular health-promoting initiatives.

### 4.2 Future development and research

Given the lack of research on group supervision using the taping method, the results of this qualitative study may contribute to increased knowledge in the field. However, further research, employing qualitative, quantitative, and mixed-method study designs, is needed. For example, more knowledge about how specific creative methods, such as taping, contribute to and influence the group supervision process is required. Do certain creative methods promote creativity, behavioral change, and health more effectively than others, or is the group supervision context itself sufficient to stimulate creativity and improved health?

The present study did not aim to compare taping with other creative supervision methods, and conclusions about its relative effectiveness cannot be drawn. Taping offers a combination of features that may distinguish it from purely verbal or image-based approaches: participants physically construct a shared visual scenario using figurines and symbols, which differs from methods relying solely on drawing or verbal imagery (13). Future research could compare taping with other creative methods to clarify whether these specific features offer distinct advantages.

Randomized studies with quantitative evaluations, where groups are assigned to supervision with or without creative methods (including taping), would be particularly valuable. Based on the discussion above regarding improvements in well-being, future studies could include stress measurements to evaluate potential effects.

Given the strengths of the taping method, particularly its ability to enhance clarity, it could also be applied in contexts beyond group supervision. For example, workgroups might benefit from using taping to clarify and structure workplace assessments. Additionally, taping could be used in conflict resolution to illustrate the parties’ perspectives on a conflict and foster mutual understanding. Another potential application is in clinical research, where taping could help individuals visualize and articulate situations or problems.

## 5 Conclusion

This study shows that group supervision using the taping method is feasible in healthcare workgroups. Group supervision was described as contributing to improved strategies for interactions with both patients and colleagues, enhanced well-being, and a better work environment characterized by increased cohesion and understanding within the workgroup.

The visualization facilitated by the taping method during supervision appears advantageous for workgroups, as it can promote a shared perspective on complex situations. It also supports learning by fostering reflection and helping participants identify processes, emotions, and behaviors that may require change. The shared visual representation also seems to create a shared language and provide a way to articulate and discuss emotions and frustrations arising in the workplace.

Visualization of a situation during group supervision with the taping method appears to strengthen memory. Combined with the emotional component of group supervision, this reinforcement may further support learning. Based on these findings, group supervision using the taping method seems to enhance self-regulated learning and shows promise as a health-promoting intervention that could help improve the work environment for individuals and teams in healthcare settings.

The findings suggest that certain conditions may support the use of taping in group supervision, such as dedicated time for supervision and group compositions that foster a sense of safety. However, further research is needed to clarify under which conditions the method is most effective.

## Data Availability

The raw data supporting the conclusions of this article will be made available by the authors, without undue reservation.
